# Summer Sores Secondary to a Hoof Crack in an Andalusian Stallion

**DOI:** 10.3390/pathogens10081038

**Published:** 2021-08-16

**Authors:** Adriana Palozzo, Donato Traversa, Giuseppe Marruchella, Gianluca Celani, Simone Morelli, Lucio Petrizzi

**Affiliations:** Faculty of Veterinary Medicine, University of Teramo, Piano d’Accio, 64100 Teramo, Italy; apalozzo@unite.it (A.P.); dtraversa@unite.it (D.T.); gmarruchella@unite.it (G.M.); gcelani@unite.it (G.C.); smorelli@unite.it (S.M.)

**Keywords:** horse, habronemosis, hoof crack

## Abstract

Cutaneous habronemosis in horses is caused by larvae of the spirurid nematodes *Habronema microstoma* and *Habronema muscae*. These lesions, also known as “summer sores’’, are often severe and disfiguring. Although *Habronema*-caused lesions at the coronary grooves have been described, cases of hoof cracks with secondary summer sores have never been reported. The present case describes clinic-pathological and surgical features of a quarter crack case complicated by cutaneous habronemosis at the dermal layers. A 15-year-old, Andalusian stallion was referred to the Veterinary Teaching Hospital of the University of Teramo because of a mass of the hoof and a severe lameness. The clinical examination revealed an exuberant granulation tissue protruding from a full thickness vertical quarter crack. The mass was surgically removed, and subjected to histopathological, microbiological, and parasitological analyses. A copromicroscopic examination was also performed. The feces scored PCR positive for *H. muscae*, while the skin for both *H. microstoma* and *H. muscae*, thus confirming the primary role of *Habronema* in causing the hoof mass. This is the first description of a hoof wall crack complicated by summer sores, with simultaneous gastric habronemosis. This case confirms that a prompt diagnosis during fly activity is imperative for an efficacious treatment and a timely prevention of disfiguring summer sores.

## 1. Introduction

Habronematidosis is a parasitic infection caused by adult and larval stages of *Habronema microstoma*, *Habronema muscae* and *Draschia megastoma* (Nematoda, Spirurida, Habronematoidea). These nematodes are transmitted by biting and secretophagous flies, e.g., *Stomoxys calcitrans* and *Musca domestica* [1]. When infective third larval stages (L3) are deposited on the nostrils and lips and swallowed by the equid, the parasites reach adulthood in the stomach and cause a gastric disease. When L3 are deposited on injured skin, wounds, or mucous membranes, they do not complete the life cycle and cause cutaneous and/or muco-cutaneous diseases [2,3,4,5]. This condition, also known as “equid summer sores”, is the most severe form, as the larvae cause a local inflammatory reaction characterized by itching, granulomatous, ulcerated and, most often non-healing, lesions [5,6]. Wounds tend to disappear spontaneously in winter, but they re-appear during fly seasons, though in some horses the lesions may evolve into non-healing granulomatous cancer-like masses which may attract more flies, leading to super-infections [2,3,5]. Summer sores occur mostly in frequently injured body parts, such as legs, sheath, coronet, medial canthus of eye, male genitalia, and ventral abdomen [7,8,9,10]. In Europe *Habronema* spp. is much more distributed than *D. megastoma*, thus gastric and (muco-)cutaneous habronemoses are the most important diseases caused by these parasites [1].

In horses, the so-called “crack” is the disruption of the hoof wall that occurs in parallel to the horn tubules and lamellae, which usually initiates at the level of the coronary band until the involvement of the full thickness of the hoof capsule [11,12,13,14]. Common causes of cracks are trauma to the coronet, pre-existing damage to the dermis due to local infections, abnormal hoof conformation, short shoes, inappropriate farrier practices or an abnormal landing pattern when the foot strikes the ground [15]. The most consistent finding in all quarter crack is a foot conformation with a sheared heel on the side of the hoof where the lesion occurs [16]. Secondary tissue infections occurring when the crack extends into the dermal layers are characterized by lameness, pain on palpation, swollen coronary band above the defect, and draining exudate from the area if a local pressure is applied [11]. Although lesions at the distal hind coronary grooves are often described [9,10], no cases of hoof wall cracks in *Habronema*-infected horses are known. Thus, the present report describes clinic-pathological and surgical features of a quarter crack case complicated by cutaneous habronemosis at the dermal layers.

## 2. Case Study

### 2.1. Case Description

A 15-year-old Andalusian stallion living in Southern Italy was referred in April 2021 to the Veterinary Teaching Hospital (VTH) of the University of Teramo (central Italy) due to the presence of a neoformation at the level of the hoof.

The owner noticed a quarter crack of the right forelimb hoof two months before the referral, and a corrective shoe (three-quarter bar shoe) was applied. However, the horse worsened in the following 20 days, showing a 4/5 lameness of the injured limb and a neoformation on the hoof crack. The referring veterinarian then treated the horse with an anti-inflammatory and antimicrobial therapy for 10 days, which was stopped 15 days before the hospitalization Despite the treatment, the owner reported a continuous growth of the lesion and referred the animal to the VTH of Teramo.

**Figure 1:**
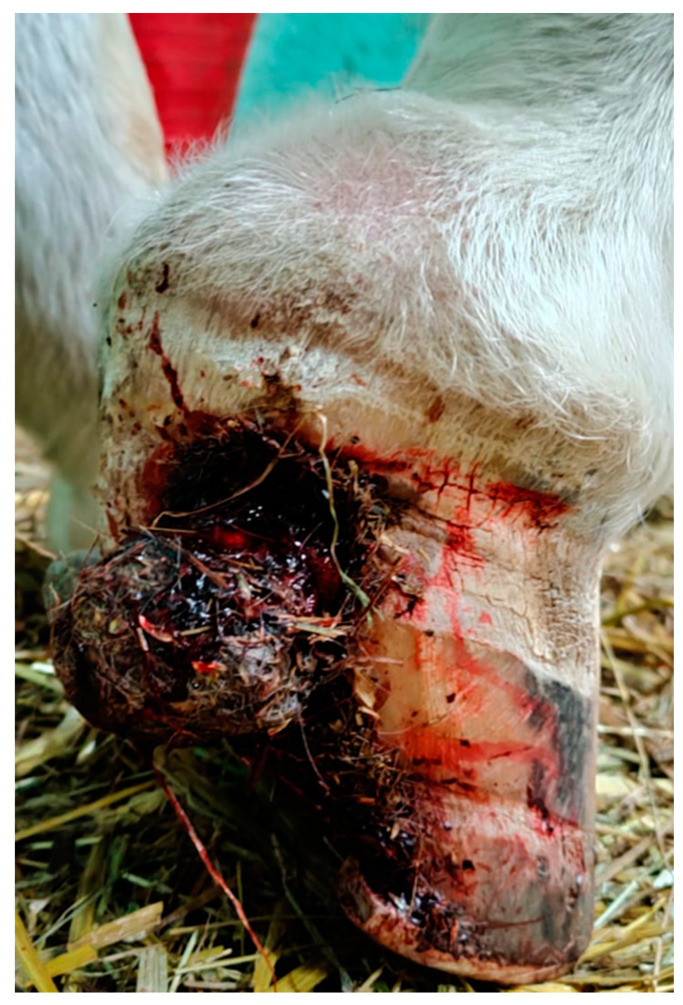
Lateral aspect of the right forelimb hoof with a bleeding full thickness vertical quarter crack and an exuberant mass protruding from the hoof crack. The mass was brown in color and had a nut-shape with hard and rough consistency.

A bleeding full thickness vertical quarter crack on the lateral aspect of the right forelimb hoof, and an exuberant granulation tissue protruding from the hoof crack, were noticed at the clinical examination. The mass was brown in color and had a nut-shape of 2 cm in diameter with hard and rough consistency (Figure 1).

A warm swelling on the coronary band and the lateral bulb of the heel of the injured leg was evident, and the horse showed pain at palpation and was 4/5 lame. No alterations of the third phalange (PIII) bone were evident at the radiographic examination.

First, standing surgery was carried out, aiming at removing the mass and performing further examinations. The mass was excised after sedation with medetomidine (7 μg/kg IV) and butorphanol (0.02 mg/kg IV). The crack was explored and widened to allow drainage and application of antiseptics. Loco-regional cephalic perfusion (right front leg) with 1 gr of gentamicin and 10 mL of lidocaine 2% was also performed, and a foot bandage was applied and changed q24h for 2 days, then q48h. A sample of the excised mass (1 cm × 1 cm × 0.4 mm) was fixed in 10% neutral buffered formalin, embedded in paraffin, and then processed for histopathological examination (hematoxylin-eosin stain, H&E). Skin scrapings obtained from the excised mass were placed in sterile tubes containing absolute ethanol for parasitological microscopic and molecular examinations. A fecal sample was also collected for copromicroscopic analysis. The swab carried out from the mass was inoculated onto blood agar and MacConkey agar and incubated at 37 °C for 24 h. Pure culture was observed on blood agar and gram stain, catalase and oxidase test were performed, yielding the identification of *Streptococcus* spp. Three days after surgery the antibiogram performed on the pure *Streptococcus* spp. culture revealed an infection susceptible to cefazoline. Thus, a loco-regional cephalic perfusion (right front leg) with 2 gr of cefazoline was performed. Furthermore, based on results of parasitological examinations (see Section 2.2) the horse was treated with one dose of moxidectin (Equest^®^ Zoetis Italia Srl, Rome, Italy) 0.4 mg/kg *per os*.

**Figure 2:**
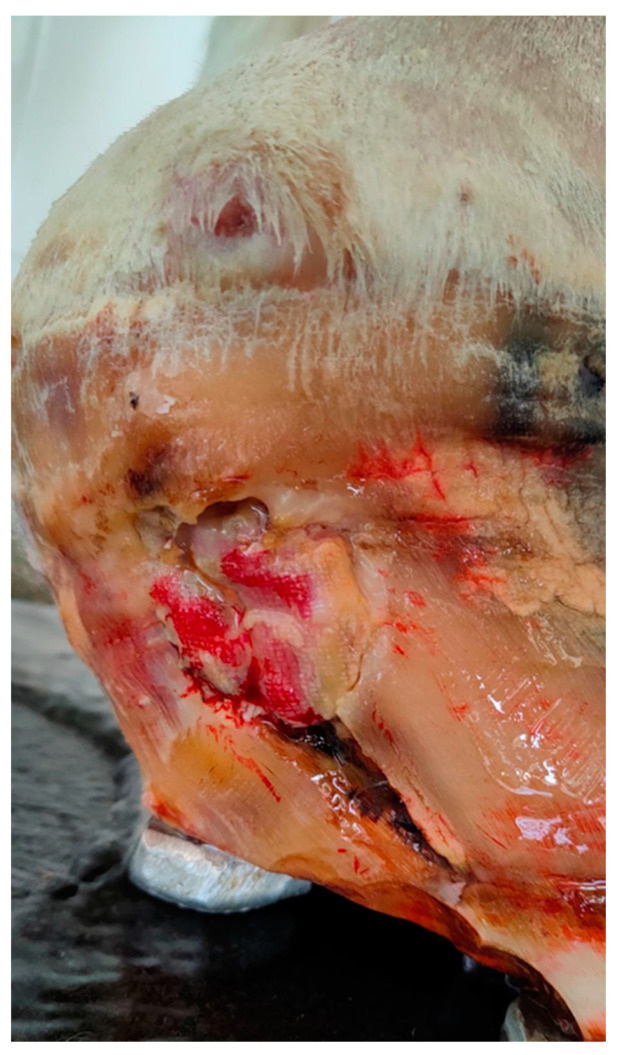
Suppurative exudate draining from the coronary band (2 weeks after the first surgery).

Two weeks after, a second surgery under general anesthesia, was carried out due the poor clinical improvement of the stallion and the presence of an exudate draining from the coronary band (Figure 2).

**Figure 3:**
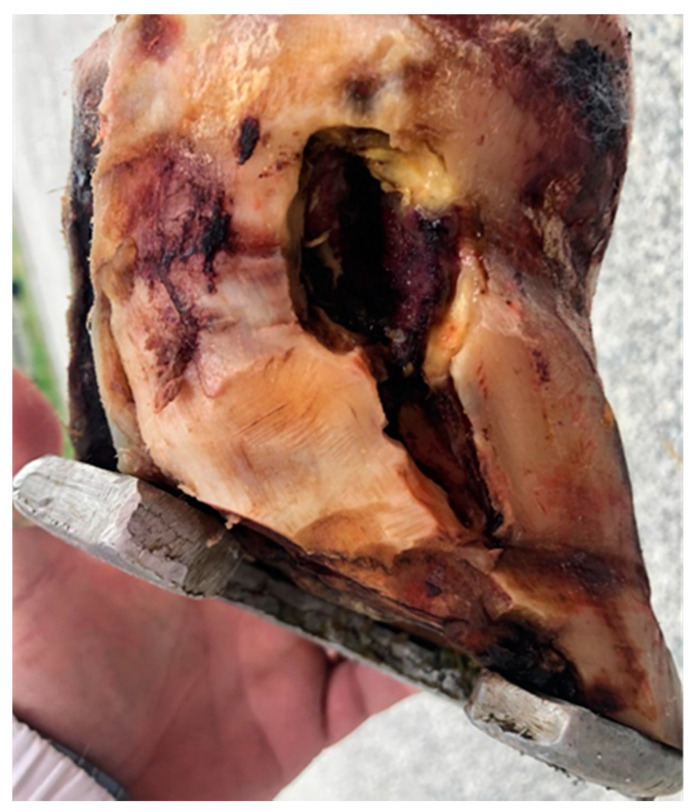
Second surgery: extensive debridement of the hoof crack.

The horse was premedicated with acepromazine (20 μg/kg IM) and sedated with medetomidine (7 μg/kg IV); general anesthesia was induced with ketamine (2.2 mg/kg IV) and diazepam (0.06 mg/kg IV) and maintained with isoflurane and a constant rate infusion of medetomidine (3.5 μg/kg/h IV). A cleansing and extensive debridement of the crack (Figure 3), with a loco-regional cephalic perfusion with 2 g of cefazoline, was performed. A foot bandage was applied and changed at 2 to 3-day intervals.

The medical management after surgery included the administration of NSAID (suxibuzone 3.75 mg/kg SID *per os*), along with antimicrobial therapy (sulfadiazine (200 mg/mL) + trimethoprim (40 mg/mL) 20 mg/kg SID *per os*) for one-week hospitalization.

Post-operative care after first and second surgery included cleaning and disinfection of the surgical site with normal saline solutions (0.9% NaCl) containing diluted povidone-iodine every two days, starting 48 h after surgery, and a constant change of hoof bandage. 

The horse recovered uneventfully, the lameness improved (3/5) and the warm swelling on the lateral bulb of the heel of the injured leg was markedly reduced. The stallion was discharged from the hospital 7 days after the second surgery.

Follow-up was obtained by telephone interviews with the owner and the referring veterinarian. The short-term follow-up reported improvement of the lameness and no recurrence of the exuberant granulomatous mass. A prosthetic hoof wall repair material was used to provide an appropriate stabilization for the quarter crack repair.

### 2.2. Parasitological Procedures

The feces were undertaken to McMaster analysis and conventional flotation procedure using a ZnSO4 solution with a specific gravity of 1.35 [17]. As previously described [18], an amount of ~500 µL of fecal supernatant was divided in two aliquots of ~100 µL and ~400 µL, the first immediately transferred onto two glass slides and examined under a light microscope (Axioscope 40, Zeiss, Oberkochen, Germany) and the other subjected to a molecular test (Section 2.2.1).

Skin scrapings from the lesion obtained during the first surgery were divided in two equivalent aliquots for subsequent microscopic and molecular examinations (Section 2.2.1). The first aliquot was processed as briefly described below [19,20]. The material was minced with a sterile blade, placed into a Petri dish, washed with physiologic saline solution, and digested with a solution of pepsin and hydrochloric acid (HCl). The sediment was then placed on a slide, covered with a glass, and examined at 100× magnification using a light microscope (Axioscope 40, Zeiss, Oberkochen, Germany).

The copromicroscopic examinations showed the presence of strongyle eggs with value < 50 eggs per gram of feces (i.e., result with no significance in the present case report), while the skin material was microscopically negative for parasite elements.

#### 2.2.1. Molecular Analysis

Stool supernatant and digested skin were subjected to DNA extraction and molecular examination as previously described using a two-step semi-nested PCR protocol able to discriminate target species-specific sequences within the rDNA ITS2 of *H. microstoma* and *H. muscae* in horse feces and skin [18,20,21]. The feces scored molecularly positive for *H. muscae*, while the skin for both *H. microstoma* and *H. muscae*.

### 2.3. Histopathology

**Figure 4:**
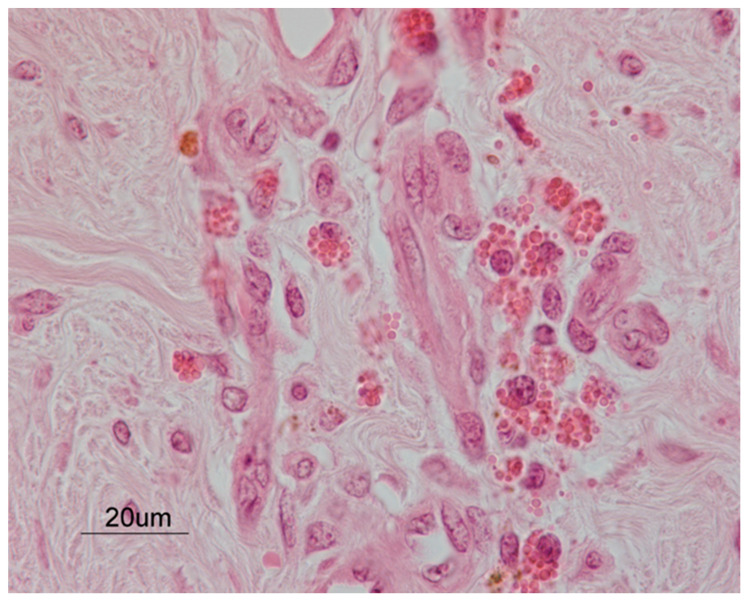
A histopathological examination of the dermal layer from the hoof lesion. A cluster of eosinophils was clearly observed, close to newly formed blood vessels. Hematoxylin and eosin stain. Final magnification 630×.

An intense inflammatory response was microscopically observed mainly at the dermal layer. More in detail, multiple foci of suppurative inflammation were seen, along with aggregates of spherical-shaped bacteria. Moreover, areas of necrosis and granulation tissue were present, along with scattered eosinophils within the dermis (Figure 4).

## 3. Discussion

In horses, the hoof capsule guarantees protection to the dermal and bone structures within the capsule [22]. Separations or defects in the hoof wall may lead to severe infections, which occur when the crack extends into the dermal layers. Most horses with hoof crack are referred for treatment between December and April each year [23]. Accordingly, in the present case the stallion was presented in March. Environmental factors (e.g., weather, ground conditions) can lead to alterations in the hoof wall structures, being responsible for the higher incidence of hoof crack in late winter–spring [24,25].

The treatment for quarter cracks relies on corrective shoeing aimed at unloading the affected wall [16,26]. In most cases, however, more interventions are necessary to treat the defect, especially if the crack is worsened by infection and lameness. In this case the corrective shoeing, the antimicrobial and nonsteroidal anti-inflammatory therapy, and the debridement of the hoof crack performed before the hospitalization have failed, likely for persistent bacterial and parasitic infections. Both crack and lameness improved after a combination of surgical treatment with extensive debridement and medical treatment including disinfection of the surgical site, loco-regional cephalic perfusions, antimicrobial and nonsteroidal anti-inflammatory therapy, and a single dose of moxidectin.

The prognosis for successful treatment of a hoof crack varies from good up to guarded [14], and the cause of the crack must be addressed to prevent recurrence [23]. The unicity of this case was the presence of the mass protruding from the hoof crack. Differential diagnosis based on the location were neoplasms, e.g., keratoma [27], squamous cell carcinoma [28] and melanoma [29], or non-neoplastic mass as keloid and exuberant granulation tissue [27]. Based on the gross appearance of the mass the differential diagnosis in the here case were primary cutaneous habronemosis, sarcoids, fibromas/fibrosarcomas, squamous cell carcinomas, haemangiomas, melanomas, papilloma, simple granulation and subcutaneous phycomycosis [6].

There is scarce information on healing process of foot wounds, though similarities exist between foot and skin [30]. Foot injuries follow the same healing processes of skin wounds, albeit differences due to (i) rigid nature and pattern of replacement of the stratum corneum, and (ii) variety of epithelial types found in the foot. Wounds involving the full thickness of the foot heal slowly, due the limited proliferation of the laminar epithelium. Hence, the defect in the wall moves distally with normal hoof wall growth [30]. Most of the complication seen in the healing of foot wounds are like those of skin wounds, while wound contraction is absent and exuberant granulation tissue is rarely observed [30]. Horses have a predisposition to produce excessive granulation tissue on wounds of the lower extremities, likely for local chronic inflammation. Accordingly, chronic inflammation must be prevented and properly treated because treatment of granulation tissue is challenging and requires the surgical excision of the protruding tissue [31].

This is the first report of hoof wall cracks in a *Habronema*-infected horse. The secondary intention healing and the persistent bacterial and parasitic infections have contributed to the exuberant granulation tissue reaction. In fact, summer sores often evolve into non-healing cancer-like masses [2] due to the local inflammation and larval death causing an allergic immune response [8,32]. Moreover, a septic pododermatitis with granulation tissue was confirmed by microbiological and histological investigations. Eosinophilia and multiple foci of coagulative necrosis [4,5] are the most common histologic changes in cutaneous habronemosis. The primary role of *Habronema* in causing the exuberant mass was then confirmed by the PCR-positivity to both *H. microstoma* and *H. muscae*, although no larvae were found at the histological examination. In fact, larvae of *Habronema* live for less than one month in the cutaneous tissue because the local allergic immune response kills them and their death triggers the appearance of exuberant, necrotic, or calcified lesions [8,32,33], that some antigens released by dead larvae will continuously act and cause those exuberant masses. The absence of larvae is not surprising and already described in PCR-positive horses [20]. Therefore, live *Habronema* larvae can only be found in very early stages of infection, though their DNA may persist and detectable upon molecular amplification [20], as here described. As the horse was referred months after the appearance of the lesion, it is argued that the infection occurred in the first months of the year.

The positivity of the horse at the molecular examination of feces (i.e., at present the most reliable approach to diagnose gastric habronemosis) further supports the primary role of *Habronema* in causing the hoof lesions and confirm previous data which showed the correlation between animals suffering from summer sores and concomitant gastric habronemosis [1,2].

Before that *Habronema*-caused lesions develop, sores usually are painful and itching, so that the infected animals bite and rub the lesions leading to auto-traumatisms. In this case it is likely that the crack attracted infected flies, which deposited the larvae which, consequently, triggered local immunity response and development of the local mass first, and the opportunistic bacterial infection, then.

## 4. Conclusions

In conclusion, this case illustrates the risk for horses affected by hoof crack to suffer from severe summer sores. The diagnosis, in this case, was untimely, as the horse was referred 20 days after that the mass appeared on the hoof crack. A molecular prompt diagnosis would have allowed a timely treatment and a faster recovery, minimizing the factual risk for a potentially disfiguring infection.

The fly season is usually spring–summer, but the current global warming is extending its duration throughout much of the year [34,35]. Thus, a prompt diagnosis of habronemosis during fly activity especially in horses with history of infection and/or living in endemic areas, is imperative for a timely treatment, and an efficacious prevention of disfiguring cutaneous habronemosis.

## Data Availability

Data sharing not applicable. No new data were created or analyzed in this study.
